# Interprofessional Insights into Shared Decision-Making in Inpatient Mental Health Facilities for Youth in Austria—A Cross-Sectional Study

**DOI:** 10.3390/healthcare14203522

**Published:** 2026-10-19

**Authors:** Miriam Subhieh, Valentina Mitgutsch, Harald Stummer

**Affiliations:** 1 Department of Health Sciences, Hochschule Campus Wien, University of Applied Sciences, 1100 Vienna, Austria; miriam.subhieh@hcw.ac.at; 2 Institute of Health Management, IMC Krems University of Applied Sciences, 3500 Krems, Austria; harald.stummer@umit-tirol.at; 3 Master’s Programme Human Medicine, Karl Landsteiner University, 3500 Krems, Austria; 4 Institute for Management and Economics in Healthcare, UMIT TIROL—Private University for Health Sciences and Health Technology, 6060 Hall in Tirol, Austria

**Keywords:** shared decision-making, child and adolescent psychiatry, interprofessional collaboration, mental healthcare, patient-centered care, healthcare professionals, Austria

## Abstract

**Highlights:**

**What are the main findings?**

- Significant differences in SDM knowledge were observed across professional groups, whereas reported SDM implementation did not differ significantly between professions.
- Professional experience was significantly associated with SDM use. Higher professional experience was associated with a lower likelihood of reporting SDM use. Organizational factors were identified as the most relevant barriers to SDM implementation.

**What are the implications of the main findings?**

- Interprofessional SDM training may help reduce profession-specific differences in SDM-related knowledge and strengthen a shared understanding of patient participation.
- Implementation efforts should focus on organizational and structural improvements, such as continuity of care and clear SDM processes, to support patient-centered decision-making in child and adolescent psychiatry.

**Abstract:**

Introduction: Shared Decision-Making (SDM) is increasingly recommended in child and adolescent psychiatry (CAP), yet little is known about its implementation across professional groups in Austrian inpatient CAP services. This study examined differences in SDM knowledge and implementation between professions and explored the association between professional experience and self-reported SDM use. Methods: A cross-sectional online survey was conducted among healthcare professionals working in Austrian inpatient CAP facilities. The questionnaire assessed SDM knowledge, attitudes, implementation, perceived barriers, and sociodemographic characteristics. Group differences were analyzed using Kruskal–Wallis tests, and binary logistic regression was used to examine the association between professional experience and SDM use. Results: A total of 56 healthcare professionals participated. Most respondents reported using SDM in clinical practice (83.9%) and expressed positive attitudes towards patient involvement. Significant differences in SDM knowledge were found across professional groups (H(3) = 8.99, *p* = 0.029), whereas SDM implementation scores did not differ significantly between professions (H(3) = 3.27, *p* = 0.352). Organizational and planning-related factors were identified as the main barriers to SDM implementation. Professional experience significantly predicted SDM use (OR = 0.45, 95% CI [0.22, 0.90], *p* = 0.025). Higher professional experience was associated with a lower likelihood of reporting SDM use. Conclusions: Participants generally reported frequent use of SDM in Austrian inpatient CAP services. While professional groups differed in SDM knowledge, reported SDM use was comparable across professions. Organizational barriers were identified as the most frequently reported obstacles to SDM implementation. Future research should examine how organizational and educational strategies may support SDM implementation in CAP settings.

## 1. Introduction

During and following the COVID-19 pandemic, mental health problems among children and adolescents increased substantially compared to pre-pandemic levels. There has been a considerable rise in the ratio of psychological stress symptoms in children and adolescents from 69.4% to 82.6% from 2020 to 2021 [1]. This increase has intensified the demand for effective and patient-centered mental health services for children and adolescents.

### 1.1. Shared Decision-Making (SDM)

Studies on participatory decision-making, commonly referred to as Shared Decision-Making (SDM), which actively involves patients in healthcare decisions, have demonstrated positive effects on treatment engagement, autonomy, and decision-making outcomes in mental healthcare [2,3]. In contrast to the model of medical paternalism, in which treatment decisions are primarily made by healthcare professionals, SDM incorporates patients’ preferences, values, and treatment goals into the decision-making process. Unlike purely informed decision-making models, SDM is characterized by a collaborative dialogue in which both healthcare professionals and patients contribute to the final treatment decision [4]. In child and adolescent psychiatry (CAP), SDM commonly involves not only healthcare professionals and young patients, but also parents or legal guardians. Consequently, treatment decisions often reflect a triadic process in which clinical expertise, the preferences of young people, and family perspectives are jointly considered [5]. Körner extended the SDM model for rehabilitation settings by distinguishing between external interactions between patients and healthcare professionals and internal interactions within multidisciplinary treatment teams [6,7]. According to this framework, effective SDM requires both internal participation within multidisciplinary teams and external participation involving patients and families. Thus, interprofessional collaboration may support, but should not be equated with SDM.

### 1.2. Interprofessional Care in CAP

Implementing SDM requires changes in organizational culture and clinical routines. However, particularly in child and adolescent mental healthcare, SDM offers opportunities for more individualized, needs-oriented, and recovery-focused care [4]. An integrative review by Gurtner et al. (2021) demonstrated that SDM implementation remains uneven across professional groups and recommended extending SDM beyond physicians to include nursing and allied health professions [7].

The relevance of this recommendation is particularly evident in child and adolescent psychiatry (CAP), where treatment is typically delivered by multidisciplinary teams. Interprofessional teams in CAP settings typically include physicians, psychologists and psychotherapists, physiotherapists, speech and language therapists, occupational therapists, social workers, and nursing staff. Additional healthcare professions represented in CAP services may include social pedagogues, dietitians, orthoptists, and other professionals involved in the multidisciplinary care of children and adolescents. These teams are structured based on the specific needs of each patient [8], a model supported by policy documents issued by the Austrian Federal Ministry of Social Affairs, Health, Care and Consumer Protection [9]. The goal of such multidisciplinary teams is to ensure high-quality care, guided by established standards and accessible services, while also safeguarding the psychosocial well-being of patients [8]. As decision-making processes in CAP frequently involve multiple professional groups, SDM cannot be understood solely from a medical perspective but requires an interprofessional perspective. According to Körner’s framework, internal participation refers to communication and decision-making processes within multidisciplinary teams, whereas external participation concerns shared decision-making between healthcare professionals and patients. In child and adolescent healthcare, this process commonly includes parents or legal caregivers alongside young patients [5,6].

In CAP, interprofessional collaboration plays a central role, as it enables comprehensive assessment, treatment planning, and continuity of care across professional groups [10]. Previous studies have shown that interprofessional collaboration is associated with improved care coordination, more positive treatment experiences, and better patient outcomes [10,11]. However, although the value of interprofessional collaboration is well-recognized, comparatively little is known about how SDM is implemented across different professional groups within child and adolescent psychiatry, particularly in Austrian inpatient settings [10].

### 1.3. Aim of the Study

Although the evidence base for SDM has expanded substantially in recent years [12,13,14], little is known about how SDM is implemented across different professional groups in Austrian child and adolescent psychiatry. In particular, evidence regarding interprofessional differences in SDM knowledge and implementation within Austrian CAP settings remains limited. Although SDM is widely recommended in mental healthcare, its implementation varies considerably across professional groups and clinical settings [7].

The aim of this study was to examine the implementation of SDM from an interprofessional perspective in Austrian inpatient child and adolescent psychiatry, psychotherapy, and psychosomatic services (CAP) [10]. A non-experimental design was adopted to address the research question. The first research question examined whether professional groups differed regarding SDM knowledge and implementation. SDM knowledge and implementation measures were employed to examine these differences. The second research question investigated whether professional experience predicted the use of SDM in clinical practice. To address this question, measures of SDM use and professional experience were included in the survey.

The findings suggest significant differences in SDM knowledge across professional groups, whereas SDM implementation appeared comparable between professions. In addition, professional experience was significantly associated with SDM use, with higher levels of professional experience being associated with a lower likelihood of reporting SDM use.

## 2. Materials and Methods

An online survey was conducted to evaluate healthcare professionals’ (physicians, nurses, psychologists, functional therapists, and social workers) perceptions regarding the integration of SDM into their daily practice within inpatient CAP facilities. Prior to data collection, the questionnaire underwent a pre-testing phase involving five individuals who either represented the target population or possessed extensive expertise in the field. The pre-test was conducted to assess the clarity, comprehensibility, relevance, and feasibility of the questionnaire.

The online questionnaire was developed based on the survey by Topp et al. (2018), which itself incorporated adaptations of the Shared Decision-Making Questionnaire for Doctors (SDM-Q-Doc) and related SDM measures, and was further tailored to the specific clinical context of child and adolescent psychiatry, psychotherapy, and psychosomatic medicine [4,15]. The questionnaire was structured according to the five domains proposed by Topp et al. (2018): (1) knowledge of SDM, (2) attitudes towards SDM, (3) implementation and use of SDM, (4) barriers to SDM implementation, and (5) sociodemographic characteristics [4]. An overview of the adapted questionnaire structure, including domains, items, response formats, and measurement levels, is provided in Appendix A.

### 2.1. Questionnaire Structure and Measures

The questionnaire consisted of open-ended, closed-ended, and mixed-format questions. Response options for closed-ended questions included single-choice, multiple-choice, and rating scale formats. Additionally, four questions were presented as item matrices, with individual statements rated using Likert scales [16]. Sociodemographic variables were primarily collected using nominal-scale items in single-choice or multiple-choice formats. Two sociodemographic variables (e.g., age and years of professional experience) were assessed as numerical open-ended responses. To ensure comprehensive representation of professional backgrounds, an additional free-text field was provided, allowing participants to specify professions not listed under the predefined categories. The questionnaire was formulated in accordance with gender-inclusive language guidelines.

The knowledge domain assessed self-reported familiarity with the concept of SDM using a rating scale and additional questions regarding prior exposure to SDM. The attitude domain evaluated beliefs regarding decision-making authority and the perceived role of patient involvement in treatment decisions. Implementation and use of SDM were assessed through questions on the self-reported application of SDM in routine clinical practice, including a dichotomous item (yes/no) that was used in the regression analysis. Further items examined the perceived usefulness and application of SDM across different diagnostic groups.

To assess SDM-related behaviors during clinical decision-making, the questionnaire included adapted items derived from the SDM-Q-Doc [15], covering key elements of the SDM process such as discussing treatment options, considering patient preferences, and reaching a shared treatment decision.

Perceived barriers to SDM implementation were assessed using a series of statements addressing organizational, professional, and patient-related factors. In addition, sociodemographic variables, including years of professional experience, were collected to investigate the association between professional experience and SDM implementation. The questionnaire was pre-tested for clarity and feasibility but was not formally psychometrically validated in the present sample.

### 2.2. Study Setting and Participants

Based on the staffing requirement model proposed by Deister et al. (2021) for Germany and considering the current structure of child and adolescent psychiatric care in Austria, an expected sample size of approximately 120–150 participants was considered feasible [8]. This estimate reflected the anticipated recruitment potential and was not based on a formal a priori sample-size calculation. To address the research questions, recruitment was restricted to the inpatient sector. The study included a total of nine child and adolescent psychiatric (CAP) inpatient departments located across different Austrian federal states. On average, each federal state has between one and three CAP institutions. At the time of data collection, no dedicated child and adolescent psychiatric inpatient institution existed in Burgenland [9,10].

All nine Austrian CAP inpatient institutions were invited to participate and were contacted through the medical directors of the respective facilities. Institutions were recruited using a convenience sampling approach. All invited institutions agreed to participate. The survey link was distributed to eligible staff members via institutional contacts; therefore, the total number of professionals who received the invitation could not be determined and a formal response rate was not calculable. Information about the study and the survey link were subsequently disseminated to eligible staff members within the institutions. Inclusion and exclusion criteria were defined prior to data collection (Table 1). Professional experience was not required for study participation. Participants who did not complete the questionnaire were excluded from the final statistical analysis.

### 2.3. Data Collection Procedure

The survey link and study information were distributed to the participating teams through the respective institutions. To maximize participation, a reminder email was sent by the principal investigator two weeks after the initial invitation. The study was conducted in accordance with the requirements of the responsible ethics committees and applicable data protection regulations. Participation was voluntary and anonymous, and all procedures complied with the General Data Protection Regulation (GDPR). Data were collected using the online survey platform UNIPARK (Tivian, Köln, Germany), a web-based survey platform for designing, administering, and managing online questionnaires. The ethics determination was received on 2 February 2023, prior to the start of recruitment. The survey remained open for six weeks, from 6 February to 20 March 2023. Following completion of the data collection, all responses were exported to IBM^®^ SPSS Statistics V29 for further analysis.

### 2.4. Statistical Analysis

The study hypotheses were examined using operationalized variables derived from the questionnaire. Due to the relatively small sample size and violations of assumptions required for parametric testing, non-parametric statistical methods were applied. To address the first research question, differences between professional groups were analyzed using the Kruskal–Wallis H test, focusing on the variables knowledge of SDM and SDM implementation score. Where significant group differences were identified, pairwise post hoc comparisons were conducted with Bonferroni-adjusted significance levels to control for multiple testing. To address the second research question, which examined the association between work experience and the binary outcome variable use of SDM (yes/no), binary logistic regression analysis was performed. In addition to significance testing, effect sizes were calculated to support the interpretation of the practical relevance of the findings. Effect sizes were interpreted according to Cohen’ s conventional benchmarks [17].

## 3. Results

The following section presents descriptive findings on the study sample and SDM-related variables, followed by analyses of professional group differences and predictors of SDM use.

### 3.1. Sample Characteristics

Of the 94 individuals who started the survey, 56 completed questionnaires were available for analysis. Incomplete questionnaires were excluded from the statistical analysis. Participants represented a multidisciplinary CAP workforce and completed the survey in an average of 10 min and 11 s. Most respondents were female (78.2%), while 21.8% were male. Professional backgrounds included physicians (26.7%), psychologists/-therapists (i.e., psychologists and psychotherapists) (21.4%), nurses (19.6%), physiotherapists (8.9%), occupational therapists (4.0%), social workers (3.6%), and other healthcare professions (8.9%). Two participants reported more than one profession. Information on profession was missing for six participants (10.7%).

Most participants were employed in acute care hospitals (*n* = 31), followed by rehabilitation clinics (*n* = 22). Nine respondents selected the category “other”, and six reported working in both an inpatient and an additional outpatient setting. Regarding geographical distribution, most participants worked in Lower Austria (51.8%), followed by Upper Austria (19.6%), Vienna (12.7%), and Tyrol (10.7%). Participants from Styria and Vorarlberg were also represented, whereas no participants were recruited from Salzburg, Burgenland, or Carinthia. Regarding professional experience, 33.9% reported up to five years of experience, while 26.8% had between six and fifteen years of experience. Approximately 39% had more than 15 years of professional experience. Detailed sample characteristics are presented in Table 2.

### 3.2. SDM Practices, Attitudes, and Perceived Barriers

Most participants reported using SDM in their clinical practice (83.9%), whereas 16.1% indicated that they did not routinely use SDM. Regarding attitudes toward treatment decision-making, more than half of the respondents (55.4%) endorsed a shared decision-making approach, indicating that treatment decisions should be made jointly by clinicians and patients. Another 26.8% reported that clinicians should make the final decision while considering patient preferences. In contrast, only a small proportion believed that treatment decisions should be made exclusively by the clinician (3.6%) or entirely by the patient (1.8%).

Complete responses across all diagnosis-specific SDM items were available for 52 participants (92.9%), whereas 4 participants (7.1%) had at least one missing value.

The reported use of SDM varied across diagnostic groups. The highest SDM scores were observed for acute and post-traumatic stress disorders (M = 4.61, SD = 1.07), anxiety disorders (M = 4.56), and psychosomatic disorders (M = 4.51). Lower scores were reported for disruptive behavior disorders (M = 4.09, SD = 1.12) and personality disorders (M = 4.19). Regarding barriers to SDM implementation, respondents reported organizational and structural factors (e.g., frequent therapist changes) as the most relevant obstacle (M = 3.07, SD = 0.56). Time constraints showed a moderate level of agreement (M = 2.73, SD = 1.54), whereas participants largely disagreed with the statement that involving patients offers no benefit (M = 1.34, SD = 0.79). A detailed overview of SDM-related variables is presented in Table 3.

### 3.3. Professional Group Differences in SDM Knowledge and SDM Implementation

To examine whether SDM knowledge and SDM implementation differed between professional groups, Kruskal–Wallis tests were conducted. The professional group comparisons were based on a subsample of 43 participants. Six participants had missing profession data, two participants reported social work as their primary profession, and five participants belonged to the category “other healthcare professions”. These categories were not included in the four-group comparison because of their small numbers. Two participants reported more than one profession and were assigned to the professional category that best reflected their primary clinical role, following the classification procedure used in the original study dataset. One participant reporting medicine and psychology was classified as psychology, and one participant reporting nursing and psychology was classified as psychology. Descriptive statistics are presented in Table 4.

**Figure 1:**
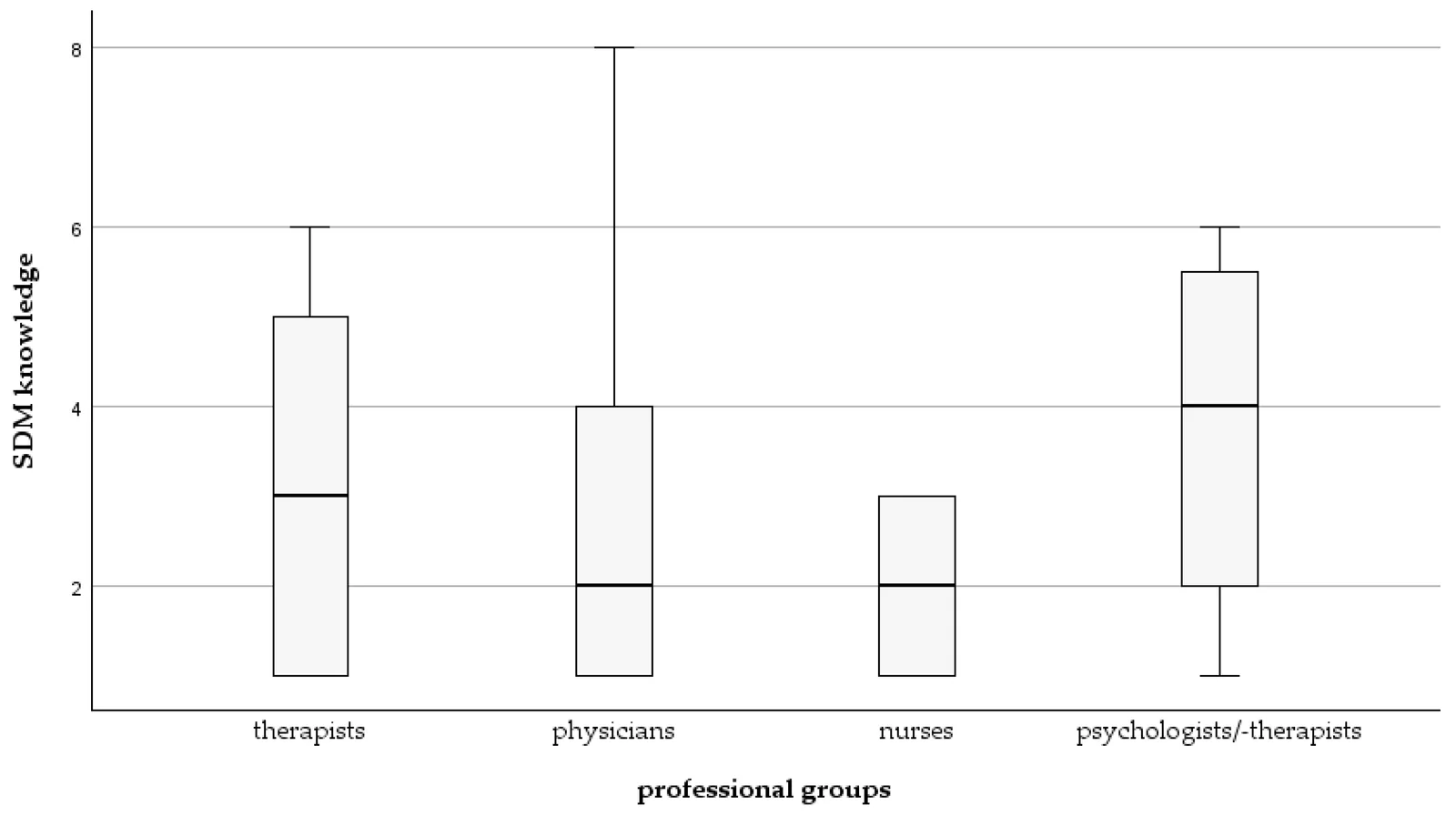
Distribution of SDM knowledge scores across professional groups.

Figure 1 shows the distribution of SDM knowledge scores across professional groups. A Kruskal–Wallis H-test revealed significant differences in SDM knowledge across professional groups, H(3) = 8.99, *p* = 0.029 (exact *p* = 0.024). Mean ranks differed across professional groups, with psychologists/-therapists (mean rank = 29.00) and therapists (mean rank = 25.43) showing numerically higher ranks than physicians (mean rank = 18.00) and nurses (mean rank = 16.80). However, these differences should be interpreted cautiously in light of the non-significant post hoc comparisons.

Although pairwise Dunn–Bonferroni comparisons did not reach statistical significance (all adjusted *p* > 0.05), the largest numerical differences were observed between psychologists and nurses (adjusted *p* = 0.074) and between psychologists and physicians (adjusted *p* = 0.084). Corresponding effect sizes indicated moderate differences (r = 0.38 and r = 0.37, respectively). The remaining pairwise comparisons were associated with small effect sizes (r = 0.04–0.23). Detailed post hoc comparisons are presented in Table 5.

**Figure 2:**
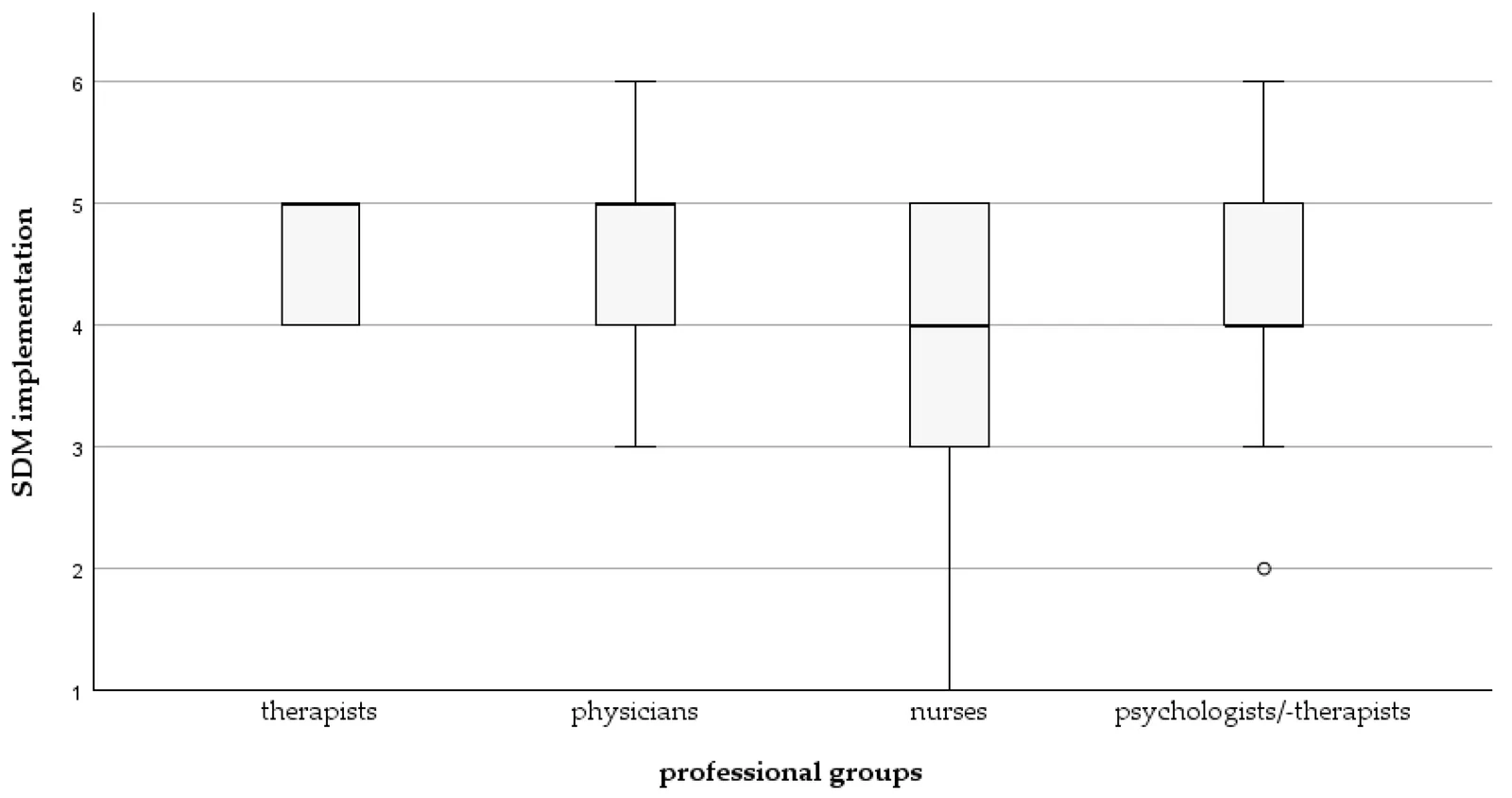
Distribution of SDM implementation scores across professional groups.

In contrast, no significant differences in SDM implementation scores were observed across professional groups, H(3) = 3.27, *p* = 0.352. Although therapists and physicians reported slightly higher mean SDM implementation scores than nurses and psychologists as illustrated in Figure 2, these differences were not statistically significant. The corresponding effect size was η^2^ = 0.01, indicating a negligible effect. Consistently, pairwise post hoc comparisons with Bonferroni correction did not reveal any significant differences between groups (all adjusted *p* > 0.05).

### 3.4. Professional Experience as a Predictor of SDM Use

**Figure 3:**
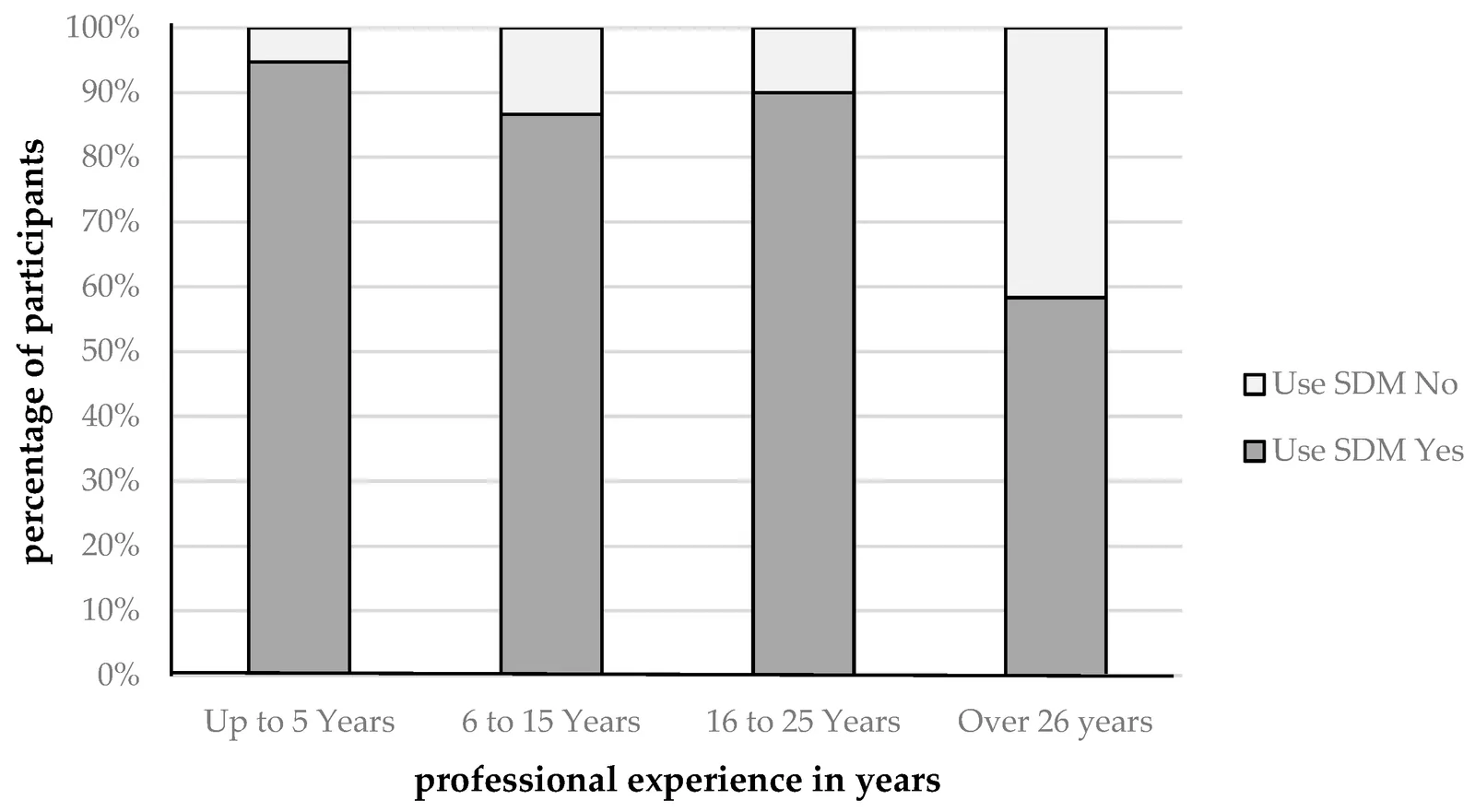
Distribution of SDM use (yes/no) across categories of professional experience.

To investigate whether professional experience predicts the use of SDM, a binary logistic regression analysis was conducted. The outcome variable in the logistic regression analysis was self-reported SDM use (yes/no). The overall model was statistically significant, χ^2^ (1) = 5.89, *p* = 0.015, indicating that professional experience contributed significantly to the prediction of SDM use. However, the results should be interpreted with caution, as only nine participants reported non-use of SDM, resulting in a highly unbalanced outcome distribution and limited numbers of outcome events for regression analysis. The model explained a small proportion of variance (Cox & Snell R^2^ = 0.10; Nagelkerke R^2^ = 0.17). Professional experience emerged as a significant predictor of SDM use (B = −0.81, SE = 0.36, *p* = 0.025). The odds ratio (OR = 0.45, 95% CI [0.22, 0.90]) indicates that increasing professional experience was associated with a lower likelihood of reporting SDM use. As illustrated in Figure 3, the proportion of SDM users exceeded that of non-users across all professional experience categories. However, the distribution of SDM use varied between experience groups, supporting the observed association between greater professional experience and lower self-reported SDM use.

The model correctly classified 83.9% of cases. However, classification performance should be interpreted with caution, as the model did not correctly identify non-users of SDM. This finding is likely attributable to the unequal distribution of the outcome variable, with most respondents reporting SDM use. Therefore, the regression findings should be regarded as exploratory. Descriptive frequencies and regression results for SDM users and non-users by professional experience are presented in Table 6.

## 4. Discussion

The present study examined the implementation of Shared Decision-Making (SDM) from an interprofessional perspective in Austrian inpatient child and adolescent psychiatry, psychotherapy, and psychosomatic services (CAP). Three principal findings emerged. First, SDM was widely reported, and participants expressed generally positive attitudes towards patient involvement in treatment decisions. Second, significant differences in SDM knowledge were observed between professional groups, whereas no significant differences were found regarding SDM implementation. Third, professional experience emerged as a significant predictor of SDM use, with more experienced professionals reporting lower levels of SDM use.

### 4.1. Professional Group Differences in SDM Knowledge and Implementation

Significant differences in SDM knowledge were observed across professional groups. However, none of the pairwise comparisons remained statistically significant after correction for multiple testing. Therefore, profession-specific differences should be interpreted cautiously. Although some pairwise comparisons showed moderate effect sizes, the absence of statistically significant post hoc differences limits conclusions regarding specific profession-level differences.

Previous research has reported an uneven implementation of SDM across professional groups in mental healthcare [7]. The present findings partially support this observation, as differences were identified in SDM-related knowledge but not in reported SDM implementation. This suggests that theoretical understanding of SDM may vary between professions, whereas clinical practice in CAP appears to be more homogeneous. The absence of significant differences in SDM implementation scores may reflect the well-established culture of interprofessional collaboration within CAP settings. Interdisciplinary teamwork, joint treatment planning, and shared responsibility for patient care are central characteristics of CAP services [10,11]. Consequently, profession-specific differences in SDM-related practices may become less pronounced than in healthcare settings where collaboration between professional groups is less integrated. In addition, the relatively small and partly unbalanced group sizes may have reduced the statistical power to detect existing differences.

The high reported level of SDM implementation is broadly consistent with current recommendations emphasizing SDM as a core component of child and adolescent mental healthcare [2,3,18,19,20,21,22]. However, the high proportion of positive responses (83.9%) may also reflect socially desirable responding [23,24]. Although social desirability cannot be entirely excluded, the anonymous online survey design was intended to minimize this potential source of bias. At the same time, the high endorsement of SDM may also indicate the genuine integration of SDM principles into routine CAP practice, where collaborative and family-centered approaches have long been established. From a theoretical perspective, the findings may further support Körner’s SDM framework, which emphasizes both internal decision-making processes within multidisciplinary teams and external decision-making processes between healthcare professionals and patients [5]. The strong interprofessional culture in CAP may facilitate these internal SDM processes and thereby contribute to more consistent patient involvement across professional groups [6,10,18,19,25]. At the same time, it is important to distinguish between team-based collaboration among professionals and SDM with patients and families. The present study focused on healthcare professionals’ perspectives and therefore cannot determine the extent to which young patients and parents experienced actual participation in decision-making. Given that SDM knowledge differed between professional groups while SDM implementation scores remained comparable, knowledge alone may not be sufficient to explain SDM implementation in everyday clinical practice. Organizational factors and team culture may play an equally important role.

### 4.2. Professional Experience and SDM Use

The logistic regression analysis demonstrated a significant association between professional experience and SDM use. Professionals with greater work experience were less likely to report using SDM in their clinical practice. This finding may indicate cohort effects in professional education and training. Given the small number of participants reporting non-use of SDM, this finding should be considered exploratory and interpreted with caution. At the same time, previous research suggests that professional experience may not be the only factor influencing SDM implementation. Topp et al. (2018) found that younger physiotherapists reported greater knowledge of SDM than their more experienced colleagues, indicating that exposure to SDM concepts during professional education may also play an important role in supporting SDM implementation [4].

Consistent with this finding, descriptive analyses indicated that the highest proportion of non-users was found among professionals with more than 26 years of experience. This pattern may reflect cohort effects or differences in professional socialization. Younger professionals may have been exposed more extensively to SDM concepts during their education and training, whereas more experienced clinicians may have been trained within healthcare systems that emphasized different decision-making models. Future research should investigate these mechanisms more closely.

The finding that professional experience was associated with SDM use has practical relevance. Training and continuing education initiatives may benefit from tailoring SDM-related content to the needs of professionals at different career stages. In particular, highly experienced professionals may benefit from practice-oriented SDM training, peer-learning opportunities, and support in integrating SDM into established clinical workflows.

### 4.3. Implications for Clinical Practice

The findings have several implications for CAP services. First, the observed differences in SDM knowledge suggest that interprofessional SDM training could help establish a shared understanding of patient participation across professional groups. Such training may contribute to reducing profession-specific differences in SDM-related competencies.

An important finding of this study was that the most relevant barriers to SDM implementation were organizational and planning-related rather than attitudinal. Participants largely disagreed with statements questioning the benefit of patient involvement, while organizational factors such as continuity of care, therapist turnover, and planning processes were identified as important obstacles. This suggests that future implementation efforts should focus not only on SDM education but also on structural and organizational improvements within CAP services.

The present findings indicate that system-level interventions may be at least as important as educational approaches. Improving continuity of care, strengthening interdisciplinary communication, establishing clear SDM procedures, and optimizing organizational workflows may represent particularly promising strategies for supporting SDM implementation. The present findings suggest that organizational factors may represent an important target for SDM implementation efforts. Future studies should examine whether organizational interventions lead to greater improvements in SDM implementation than educational approaches alone. Finally, the high overall acceptance of SDM across professional groups demonstrates the potential for embedding SDM more systematically within CAP services and interprofessional treatment pathways.

### 4.4. Strengths and Limitations

A key strength of this study is its contribution to an under-researched field, providing initial empirical insights into the implementation of SDM across different professional groups in Austrian child and adolescent psychiatry. Another strength is the inclusion of multiple professional groups, allowing for an explicitly interprofessional perspective on SDM implementation. Furthermore, the anonymous online survey design enabled broad geographical recruitment while minimizing social pressure during data collection.

Several limitations should be considered when interpreting the findings. First, the adapted questionnaire was based on previously published instruments and survey approaches but was not formally psychometrically validated in the present sample. Consequently, the results should be interpreted with caution, particularly regarding measurement accuracy and construct validity. Nevertheless, a pre-test involving representatives of the target population and individuals with subject-matter expertise was conducted prior to data collection to assess the questionnaire’s clarity, comprehensibility, relevance, and feasibility. Second, the sample size achieved remained below the a priori target, which may have limited statistical power and contributed to the absence of significant post hoc group differences. The study should therefore be regarded as exploratory. The sample size achieved was substantially smaller than the anticipated recruitment target, and several professional subgroups were represented by only a small number of participants. Consequently, statistical power was limited, and uncertainty in both the professional-group comparisons and the regression analysis should be considered when interpreting the findings. Third, some professional groups, particularly the therapist subgroup, were comparatively small, limiting the interpretation of profession-specific findings. Fourth, the cross-sectional design does not allow for causal conclusions regarding the relationship between professional experience and SDM use. Fifth, all data were based on self-report measures and may therefore be affected by response bias or social desirability.

Furthermore, the logistic regression model showed limited discriminatory performance due to the unequal distribution of SDM users and non-users within the sample. Only nine participants reported non-use of SDM, resulting in a small number of outcome events for regression analysis. Consequently, the regression coefficients may be unstable, and the findings should be interpreted as exploratory rather than confirmatory. Future studies with larger samples and a more balanced distribution of outcome categories should examine these associations using more robust modeling approaches.

Finally, although geographical coverage across Austria was generally satisfactory, the findings may not be fully representative of all CAP settings, particularly outpatient and community-based services.

### 4.5. Future Research

Future research should include larger and more balanced samples across professional groups and institutions to improve statistical power and representativeness. Expanding recruitment to outpatient services, home-treatment programs, and community mental health settings may provide a more comprehensive understanding of SDM implementation across the continuum of care.

Future implementation studies should examine whether organizational interventions, such as improving continuity of care, reducing therapist turnover, or introducing standardized SDM workflows, lead to greater improvements in SDM implementation than educational approaches alone. The neutral rating of time constraints also suggests that future studies should investigate under which circumstances time becomes a relevant barrier and which workflow modifications may facilitate SDM in routine clinical practice.

In addition, future studies should investigate practitioner–patient SDM processes directly rather than relying exclusively on healthcare professionals’ self-reports. Including the perspectives of children, adolescents, and parents would provide valuable insights into how SDM is experienced by service users and whether reported SDM practices correspond to patients’ experiences.

Finally, the descriptive findings suggest potential differences in SDM use across diagnostic groups. Future studies should examine whether diagnosis-specific characteristics influence opportunities, barriers, and attitudes towards SDM implementation in CAP settings. Longitudinal studies may further help clarify how professional experience influences SDM use over time and whether targeted SDM interventions can strengthen implementation across different professional groups and career stages.

## 5. Conclusions

This study provides novel insights into the implementation of Shared Decision-Making (SDM) from an interprofessional perspective in Austrian child and adolescent psychiatry. While significant differences in SDM knowledge were observed across professional groups, reported SDM implementation was consistently high and did not differ significantly between professions. These findings indicate that healthcare professionals in this sample reported the frequent use of SDM across professional groups.

Importantly, organizational and planning-related factors emerged as the most relevant barriers to SDM implementation, whereas negative attitudes toward SDM were rarely reported. This indicates that future implementation efforts should focus not only on professional education but also on structural improvements such as continuity of care, interdisciplinary communication, and clear SDM processes.

Furthermore, professional experience was associated with SDM use, with greater professional experience being associated with a lower likelihood of reporting SDM use, highlighting the need for tailored implementation and training strategies across different career stages. Given the exploratory nature of the study and the relatively small and unbalanced sample, the findings should be interpreted with appropriate caution and verified in larger studies. Overall, the findings contribute to a better understanding of SDM implementation in CAP and provide a foundation for future interventions aimed at strengthening patient-centered decision-making in mental healthcare.

## Figures and Tables

**Table 1:** Eligibility criteria for participants.

Category	Inclusion Criteria	Exclusion Criteria
Profession	Physicians, nurses, physiotherapists, occupational therapists, psychologists, speech and language therapists, social workers, and other eligible healthcare professions (e.g., social pedagogy, dietetics, orthoptics)	Professionals not employed in eligible healthcare professions within CAP services
Employment status	Currently employed or self-employed in an inpatient CAP facility	Interns, trainees, retired professionals
Language proficiency	Sufficient proficiency in German to understand and complete the questionnaire	Insufficient German language proficiency
Institution type	Inpatient child and adolescent psychiatry (CAP) facilities	Outpatient facilities or non-CAP settings
Geographical criterion	Employment in one of the nine eligible CAP inpatient institutions in Austria	Employment outside the eligible institutions
Survey completion	Completed questionnaire	Incomplete questionnaire

**Table 2:** Sociodemographic and professional characteristics of the study sample.

Variables	Values	Missing
**Years of professional experience**		0.0%
0–5 years	33.9%	
6–15 years	26.8%	
16–25 years	17.9%	
26+ years	21.4%	
**Sex**		0.0%
Female	78.2%	
Male	21.8%	
Diverse	0.0%	
**Profession**		10.7%
Physicians	26.8%	
Nurses	19.6%	
Psychologists/-therapists	21.4%	
Physiotherapists	8.9%	
Occupational Therapists	3.6%	
Social Workers	3.6%	
Other Healthcare Professions *	8.9%	
**Institution location**		0.0%
Lower Austria	51.8%	
Upper Austria	19.6%	
Vienna	12.7%	
Tyrol	10.7%	
Styria	3.6%	
Vorarlberg	1.8%	

* Other healthcare professions include social pedagogy, dietetics, orthoptics, and other professions working in CAP. Note: Percentages for profession may exceed 100% due to multiple responses.

**Table 3:** SDM practices, attitudes, diagnosis-specific SDM use, and perceived barriers.

Variables	Values	Missing
**Use of SDM**		0.0%
Yes	83.9%	
No	16.1%	
**Attitude towards SDM**		0.0%
Clinician decides	3.6%	
Clinician decides considering patient preferences	26.8%	
Shared decision between clinician and patient	55.4%	
Patient decides after clinician recommendation	12.5%	
Patient decides independently	1.8%	
**Diagnosis-specific SDM use**		7.1%
Anxiety disorder	M = 4.56 (SD = 1.10)	
Acute and post-traumatic stress disorder	M = 4.61 (SD = 1.07)	
Depressive disorders	M = 4.27 (SD = 1.15)	
Obsessive-compulsive disorders	M = 4.22 (SD = 1.26)	
Disruptive behaviour disorders	M = 4.09 (SD = 1.123)	
Personality disorders	M = 4.19 (SD = 1.30)	
Psychosomatic disorders	M = 4.51 (SD = 1.10)	
**Perceived barriers to SDM implementation**		0.0%
Insufficient time resources	M = 2.73 (SD = 1.54)	
No perceived benefit of SDM	M = 1.34 (SD = 0.80)	
Organizational barriers	M = 3.07 (SD = 0.56)	

**Table 4:** Professional group differences in SDM knowledge and SDM implementation scores.

Profession	*N* (%)	SDM Knowledge M (SD)	SDM Implementation M (SD)
Therapists	7 (16.3)	3.14 (2.19)	4.57 (0.54)
Physicians	14 (32.6)	2.93 (2.24)	4.50 (0.86)
Nurses	10 (23.3)	1.90 (0.88)	3.80 (1.23)
Psychologists/-therapists	12 (27.9)	3.75 (2.01)	4.17 (1.03)
*p*-Value *		0.029 *	0.352

* *p* < 0.05. Note: Analyses were based on participants assigned to one of four professional groups: physicians, nurses, psychologists/-therapists, and therapists (physiotherapists and occupational therapists; *n* = 43).

**Table 5:** Pairwise Dunn–Bonferroni post hoc comparisons for SDM knowledge across professional groups.

	z	*p*	r
Nurses-Physicians	0.255	1	0.04
Nurses-Therapists	1.538	0.744	0.23
Nurses-Psychologists/-therapists	−2.504	0.074	0.38
Physicians-Therapists	1.41	0.951	0.22
Physicians-Psychologists/-therapists	−2.457	0.084	0.37
Therapists-Psychologists/-therapists	−0.66	1	0.10

**Table 6:** Logistic regression analysis of professional experience as a predictor of SDM use.

Professional Experience	Yes (%)	No (%)	Total (%)
≤5 years	32.1	1.8	33.9
6–15 years	23.2	3.6	26.8
16–25 years	16.1	1.8	17.9
26+ years	12.5	8.9	21.4
Total	83.9	16.1	100.0
*p*-Value			0.025

Note: Model fit: χ^2^ (1) = 5.89, *p* = 0.015; Cox & Snell R^2^ = 0.10; Nagelkerke R^2^ = 0.17.

## Data Availability

Owing to institutional restrictions, the raw data are not publicly available. The study data contains information collected from healthcare professionals working in highly specialized settings, and public sharing could potentially compromise confidentiality obligations. Therefore, access to the raw dataset is restricted in accordance with institutional requirements and data protection considerations.

## Abbreviations

The following abbreviations are used in this manuscript:

CAP	Child and Adolescent Psychiatry
GDPR	General Data Protection Regulation
SDM	Shared Decision-Making
SDM-Q-Doc	Shared Decision-Making Questionnaire for Doctors

## Appendix A

The questionnaire was adapted from the survey developed by Janine Topp et al. (2018) and further modified for the context of Austrian inpatient child and adolescent psychiatry, psychotherapy, and psychosomatic services [4]. The instrument comprised five domains: knowledge of SDM, attitudes towards SDM, implementation and use of SDM, barriers to SDM implementation, and sociodemographic characteristics. The adapted questionnaire structure can be found in Table A1.

**Table A1:** Adapted questionnaire structure.

Domain	Main Item(s)	Scale Level	Response Format	Number of Items
Knowledge	How much do you know about the concept of Shared Decision-Making (SDM)?	Ordinal	Likert scale	1
	In what context did you learn about Shared Decision-Making?	Nominal	Multiple choice + open response	1
Attitudes	Who do you think should make treatment decisions?	Nominal	Single choice	1
	“I consider Shared Decision-Making useful for treatment planning in patients with…”	Ordinal	Item matrix (Likert scale) + open response option	8
SDM Implementation/Use	Do you use Shared Decision-Making in your daily clinical practice with children and adolescents?	Binary	Single choice (Yes/No)	1
	“I frequently apply the concept of Shared Decision-Making.”	Ordinal	Likert scale	1
	Who usually makes treatment decisions in your daily clinical practice?	Nominal	Single choice	1
	“Shared Decision-Making is applied in my daily practice with patients with…”	Ordinal	Item matrix (Likert scale) + open response option	8
	Please think about the last treatment in which a treatment decision was made.	Open-ended	Free text response	2
	“The following statements refer to the treatment situation described above.”	Ordinal	Item matrix (Likert scale)	9
Barriers to SDM Implementation	“The following factors may hinder the implementation of Shared Decision-Making.”	Ordinal	Item matrix (Likert scale) + open response option	10
Sociodemographic Characteristics	Current profession	Nominal	Single choice + open response option	1
	Sex	Nominal	Single choice	1
	Professional education/training pathway	Nominal	Multiple choice	1
	Federal state of employment	Nominal	Single choice	1
	Years of professional experience	Nominal	Single choice	1
	Weekly working hours	Metric	Open-ended response	1
	Average treatment/session duration	Metric	Open-ended response	1
	Patient groups treated in daily practice	Nominal	Multiple choice + open response option	1
	Type of institution	Nominal	Single choice + open response option	1
